# Targeting Inflammation and Regeneration: Scaffolds, Extracellular Vesicles, and Nanotechnologies as Cell-Free Dual-Target Therapeutic Strategies

**DOI:** 10.3390/ijms232213796

**Published:** 2022-11-09

**Authors:** Maria Peshkova, Nastasia Kosheleva, Anastasia Shpichka, Stefka Radenska-Lopovok, Dmitry Telyshev, Alexey Lychagin, Fangzhou Li, Peter Timashev, Xing-Jie Liang

**Affiliations:** 1 World-Class Research Center “Digital Biodesign and Personalized Healthcare”, Sechenov University, 119991 Moscow, Russia; 2 Institute for Regenerative Medicine, Sechenov University, 119991 Moscow, Russia; 3 Laboratory of Clinical Smart Nanotechnologies, Institute for Regenerative Medicine, Sechenov University, 119991 Moscow, Russia; 4 FSBSI Institute of General Pathology and Pathophysiology, 125315 Moscow, Russia; 5 Chemistry Department, Lomonosov Moscow State University, 119991 Moscow, Russia; 6 Institute for Clinical Morphology and Digital Pathology, Sechenov University, 119991 Moscow, Russia; 7 Institute of Biomedical Systems, National Research University of Electronic Technology, 124498 Moscow, Russia; 8 Institute of Bionic Technologies and Engineering, Sechenov University, 119991 Moscow, Russia; 9 Department of Traumatology, Orthopedics and Disaster Surgery, Sechenov University, 119991 Moscow, Russia; 10 CAS Key Laboratory for Biomedical Effects of Nanomaterials and Nanosafety, CAS Center for Excellence in Nanoscience, National Center for Nanoscience and Technology of China, Beijing 100190, China; 11 University of Chinese Academy of Sciences, Beijing 100049, China

**Keywords:** osteoarthritis, inflammation, synovitis, cell-free tissue engineering, extracellular vesicles, matrix-bound nanovesicles

## Abstract

Osteoarthritis (OA) affects over 250 million people worldwide and despite various existing treatment strategies still has no cure. It is a multifactorial disease characterized by cartilage loss and low-grade synovial inflammation. Focusing on these two targets together could be the key to developing currently missing disease-modifying OA drugs (DMOADs). This review aims to discuss the latest cell-free techniques applied in cartilage tissue regeneration, since they can provide a more controllable approach to inflammation management than the cell-based ones. Scaffolds, extracellular vesicles, and nanocarriers can be used to suppress inflammation, but they can also act as immunomodulatory agents. This is consistent with the latest tissue engineering paradigm, postulating a moderate, controllable inflammatory reaction to be beneficial for tissue remodeling and successful regeneration.

## 1. Introduction

Osteoarthritis (OA) has long been [1,2,3] and is sometimes still [4,5] referred to as a non-inflammatory condition. Moreover, when studying “classic” inflammatory arthritides, such as rheumatoid arthritis (RA) or spondyloarthritis, researchers would often use tissue and biologic fluid samples from OA patients as negative (non-inflammatory) controls [6,7]. Although currently researchers agree that many aspects contribute to OA progression, including gender [8], it has long been regarded mainly as the consequence of cartilage wear and tear. Cartilage damage was reported to lead to joint biomechanics impairment resulting in further cartilage loss and joint deformity, while the inflammatory component was commonly underestimated.

Cartilage tissue itself, being avascular, cannot develop a classic immune response. However, when considering the joint as a whole, including synovium, ligaments, and subchondral bone, inflammation (synovitis) seems to play an important role in OA progression. There is evidence that synovitis is associated with increased OA severity [9,10], and therefore it is a promising target for currently missing disease-modifying OA drug (DMOADs) development. Most available OA management options, including lifestyle changes, pharmacotherapy, and surgery [11,12,13], also target joint inflammation to varying degrees.

Lifestyle modification is the basis of most chronic disease management and can be beneficial to compliant patients. A balanced diet and physical activity can postpone the OA onset or slow down its progression both by reducing the joint loading due to weight loss and decreasing the levels of adipokines, which are known to contribute to the inflammatory component of OA development [14,15]. Currently recommended pharmacological treatments, including non-steroidal anti-inflammatory drugs (NSAIDs) and corticosteroids, target inflammation and relieve pain [11], while the operative approaches aim to both reduce pain and improve joint functions [13]. However, one of the major drawbacks of all these strategies is that none of them address the problem of cartilage loss. Due to its low self-healing capacity, hyaline cartilage can only be repaired with involvement of cartilage-repair techniques, and various strategies, both scaffold-based and scaffold-free and cell-based and cell-free, are being proposed [16,17]. Unfortunately, most conventional cartilage tissue repair techniques focus on cartilage regeneration, while their effect on joint inflammation is rarely being discussed. Apparently, in order to fully address the problem of OA, a combination of cartilage repair techniques and strategies targeting inflammation should be considered.

This narrative review aims to summarize the evolving approaches which target both inflammation and cartilage damage to treat OA. We chose to focus on cell-free techniques and selected original research articles reporting the application of those in vivo. In addition, we aimed to promote the idea of immunomodulation as a potent tool for both inflammation management and successful regeneration.

## 2. Inflammation in an OA Joint

Homeostasis in a healthy joint is maintained by the synovial intima cells, namely type A macrophage-like synoviocytes, responsible for debris phagocytosis, and type B fibroblast-like synoviocytes, which produce synovial fluid components, including hyaluronan [18]. In an OA joint the products of the cartilage extracellular matrix (ECM) degradation (thoroughly reviewed elsewhere [7]) are bound to the pattern recognition receptors (PRRs) and recognized by the innate immune system as damage/danger-associated molecular patterns (DAMPs). Other groups of DAMPs in an OA joint include plasma proteins (e.g., α1 and α2 microglobulins, fibrinogen, vitamin D-binding protein), crystals of basic calcium phosphate, calcium pyrophosphate dihydrate, and uric acid [19], and so-called alarmins [6], including HMGB1 and the S100 family of proteins. There is evidence of some DAMPs’ ability to activate the complement system [19]. Some DAMPs, for example, crystals, rather bind to cytoplasmic PRRs (e.g., NLRP3), initiating inflammasomes activation [20,21]. However, it seems that most DAMPs activate Toll-like receptors (TLRs), a large membrane-bound family of PRRs. TLRs are reported to be expressed both in the cartilage [22], being upregulated in the lesion areas [23], and in the synovium [19]. When stimulated by DAMPs, they trigger catabolic pathways in chondrocytes [23] and proinflammatory factor production by macrophages and mast cells [19]. The proinflammatory mediators can promote matrix metalloproteinases (MMP) production, directly enhancing the catabolic processes in the cartilage. They can also stimulate angiogenesis [24], increasing the influx of plasma proteins [7], which also act as DAMPs. Thus, more DAMPs are attracted to the area. Moreover, proinflammatory cytokines can boost their own production: exposure to proinflammatory cytokines promotes proinflammatory (M1) macrophage polarization, which in turn stimulates proinflammatory cytokine synthesis [25]. Thus, multiple vicious circles of further cartilage damage are established. 

Synovial macrophages are the key cells orchestrating the processes of inflammation and healing within the joint because of their capacity to exhibit different phenotypes ranging from proinflammatory (M1) to anti-inflammatory (M2). In an OA joint, macrophages are caught into a vicious circle, being constantly attracted to the site by the perpetuated cartilage degradation and proinflammatory cytokine production, infiltrating the synovium and impairing its functions, notably, synovial fluid component synthesis [26], although to a lesser extent than in RA [27]. Alterations in the OA synovial fluid contents influence its properties, for example, the lack of hyaluronan leads to its decreased viscosity and elasticity [28], resulting in less effective lubrication of the joint surfaces and promoting cartilage damage. Aiming to find a way to disrupt the vicious circle involving the synovial macrophages, some researchers study the consequences of their depletion when modeling OA. Local macrophage depletion using intra-articular injections of clodronate-loaded liposomes has demonstrated rather positive outcomes in murine models, such as reduced MMPs’ expression in the synovial tissue and decreased osteophyte formation [29,30,31]. On the contrary, systemic depletion of macrophages in CSF-1R-GFP+ macrophage Fas-induced apoptosis (MaFIA)-transgenic mice placed on a high-fat diet induced systemic inflammation. Moreover, it led to massive infiltration of CD3+ T cells and neutrophils in the synovium [32], although neutrophil infiltration is characteristic of RA [33] and is hardly ever observed in OA [27]. 

## 3. Existing Strategies for Targeting Inflammation and Cartilage Regeneration in OA

### 3.1. Inflammation

OA can affect people of any age. Accidental trauma, for example, joint ligament damage, can lead to reactive inflammation and post-traumatic OA development. Even if the articular cartilage itself has not been damaged, the inflammatory process in the joint can lead to the activation of catabolic pathways in the cartilage tissue [34].

However, OA is generally discussed in the context of older adults and elderly patients, and one cannot talk about these groups of patients without mentioning comorbidities. Unfortunately, oral NSAIDs and acetaminophen (paracetamol), traditionally used for pain management in OA, are associated with adverse side effects. Those include gastrointestinal [35] and cardiovascular [36] damage for NSAIDs, while paracetamol use may be associated with liver [37] and renal [38] damage. On the other hand, topical NSAIDs are considered a safer but still rather effective option and are strongly recommended for OA patients [12]; however, their use is not enough to reverse the OA progression. Efficacy of intra-articular injections of steroids or hyaluronic acid (HA) remains debatable [11,12]. Mixed results were obtained when targeting proinflammatory cytokines as well. For example, anakinra, a recombinant human IL-1 receptor antagonist protein, was reported to perform better than placebo in patients with severe knee injury [39], and at the same time in OA patients its effect was comparable with placebo [40]. AMG 108, a monoclonal antibody binding the IL-1 receptor, also showed moderate effectiveness in OA patients with minimal clinical benefit [41]. Similarly, anti-tumor necrosis factor alpha (TNF-α) monoclonal antibodies, such as adalimumab and infliximab, despite sporadic encouraging evidence [42,43], were reported to have limited effectiveness in hand OA patients [44,45]. 

Speaking of arthroscopic procedures, joint lavage seems to hold promise in reducing inflammation following the removal of debris from the joint cavity; however, evidence suggests that it does not provide any significant improvement either [46].

### 3.2. Cartilage Regeneration

Despite undeniable progress in the field, cartilage regeneration remains a challenge. There are multiple approaches to cartilage repair depending on the size of the defect, and unfortunately each of them has some drawbacks. For example, bone marrow stimulating techniques, such as subchondral drilling and microfracturing, lead to the formation of fibrocartilage, whose mechanical properties are inferior to those of hyaline cartilage [47]. The use of bone marrow stimulation technique with hydrogel implantation into the defect provides the appropriate environment for hyaline cartilage formation [48]. Localized co-delivery of agents inducing hyaline cartilage formation such as bone morphogenic protein 2 (BMP2) and vascular endothelial growth factor (VEGF) receptor antagonist [49] in a hydrogel has also been proposed.

Another group of approaches is based on autologous chondrocyte implantation (ACI), sometimes applied together with collagen-based scaffolds (matrix-induced ACI, or MACI), and is used for larger cartilage defects. These approaches are reported to result in repair with hyaline-like cartilage; however, those are expensive multi-stage procedures requiring long-term rehabilitation [50]. 

In contrast, autologous stem cell transplantation may be performed in one stage, has a shorter rehabilitation period, and is less expensive than ACI. However, this approach requires longer-term studies to be recommended as a first-line treatment [50].

Osteochondral autografts or allografts are used for the largest cartilage lesions [51]. The osteochondral autograft transfer system (OATS, also known as mosaiplasty) has demonstrated good clinical outcomes, but even though the grafts are harvested from low-bearing regions in the joint, donor-site morbidity cannot be fully avoided. On the other hand, allografts, taken from deceased donors, seem to solve the problem of donor-site morbidity, but despite consensual cartilage immune privilege, some histocompatibility concerns cannot be ignored [52].

## 4. Inflammation Management in Tissue Engineering

In tissue engineering inflammation is mostly regarded as an adverse effect and a challenge to overcome. All the components of the tissue engineering triad (i.e., biomaterials, cells, and biochemical factors) are therefore being discussed in the context of biocompatibility. Furthermore, various modifications promoting better engraftment as well as minimizing the undesired immune reactions are being proposed for the existing approaches.

### 4.1. Biomaterials

Both natural and synthetic biomaterials used in tissue engineering have some strong advantages and some critical issues. For example, when assessing biomechanical properties or reproducibility, synthetic biomaterials, such as polycaprolactone (PCL), poly(glycolic acid) (PGA), polylactide (PLA), or poly(lactic-co-glycolic acid) (PLGA), are superior to natural biomaterials. On the other hand, when speaking about biocompatibility, natural biomaterials take precedence. It was reported that most synthetic polymers induce considerable inflammation in vivo [53,54], while natural biomaterials such as collagen [55] or silk [56,57,58,59] cause a significantly lower immune response. Still, synthetic polymers remain attractive substrates for tissue engineering and can be functionalized to enhance their biocompatibility. For example, magnesium hydroxide nanoparticles may help to neutralize pH changes by PLGA degradation acidic products, alleviating inflammation [60].

Hydrophilicity or hydrophobicity of the scaffold is another important characteristic affecting the protein adsorption and therefore the host immune response. Most synthetic polymers are hydrophobic, which correlates with high immunogenicity, but hydrophilic molecules such as polyethylene oxide (PEO) [61], polyethylene glycol (PEG) [61], or graphene oxide (GO) [62] can be used to modify their surface chemistry [63]. There is evidence that compound scaffolds consisting of both synthetic and natural biomaterials show rather good biocompatibility, for example, collagen from micronized porcine cartilage alleviated the inflammatory effect of a PLGA scaffold in a rat model [54]. However, the best available option in terms of biocompatibility remains decellularized ECM (dECM). Not only does dECM provide a perfect microenvironment for cells, it was also repeatedly reported to have immunomodulatory properties, including the influence on the macrophages, namely their polarization to the anti-inflammatory M2 phenotype [63,64,65,66]. Unsurprisingly, many researchers try to mimic ECM properties when designing biomaterials [67].

Some researchers propose scaffold-free approaches to avoid any possible adverse immune reactions to the biomaterials. Both chondrocyte-based [68] and synovial mesenchymal stem cells (MSCs)-based [69] cell sheets were demonstrated to promote good cartilage regeneration without any undesired inflammatory response in vivo. However, although considered “scaffold-free”, both constructs contained the ECM synthesized by the cells [70], and therefore these data may speak in favor of the use of ECM as well.

### 4.2. Cells

MSCs from different sources, the cell type the most extensively used in tissue engineering, are appreciated for their low immunogenicity and certain immunosuppressive capacity [71]. However, it was reported that in the process of differentiation in vivo their immunogenicity is induced due to MHC-I and MHC-II expression [72], and there is evidence that MSCs can cause a memory T-cell response in immunocompetent hosts [73].

MSCs’ therapeutic potential has long been discussed in the context of their differentiation capacity, i.e., ability to replace damaged tissues. However, a growing body of evidence suggests that MSCs exert their regenerative effect via paracrine activity [74]. Cytokines, chemokines, and, most importantly, extracellular vesicles (EVs) secreted by MSCs are now considered the key players in MSC-based therapies. EVs are heterogeneous membrane nanoparticles providing intercellular communication. Their research and use in various fields of regenerative medicine have been boosted over the past years, and they were demonstrated to possess all the advantages of MSCs without considerable drawbacks. For example, MSC-derived EVs exhibit no risk of tumor formation and demonstrate even lower immunogenicity than MSCs [75]. Moreover, they have lower storage demands and are therefore a promising agent for biomedical product development. Summing up, this makes cell-free approaches more and more prevalent in tissue engineering, including cartilage repair [76].

### 4.3. Biochemical Factors

Cytokines and growth factors control inflammation as well as cell proliferation, migration, and differentiation, thus orchestrating tissue remodeling and regeneration. Biochemical factors are naturally synthesized by the cellular component of tissue-engineered constructs: for example, MSCs are known to produce a variety of both growth factors and cytokines [77,78]. Moreover, cell cultures can be transfected or transduced so that the cells will secrete the desired bioactive molecules. In a study conducted by Holladay and colleagues, transfected rat bone-marrow-derived MSCs over-expressing an anti-inflammatory cytokine interleukin 10 (IL-10) demonstrated a good retention rate within collagen scaffolds in vivo for up to 7 days in comparison to unmodified cells, while the number of inflammatory cells during this period was decreased [79]. However, the authors reported that IL-10 modified the MSCs’ retention rate to reduce almost to the same level as unmodified cells by day 21, with an unexpected increase in inflammatory cell number on day 7, speculating that prolonged culturing might have altered the MSCs’ phenotype to become more immunogenic. Thus, given the difficulty to control implanted cells in vivo as well as the growing popularity of cell-free approaches, scaffold loading with bioactive factors has been developed. For example, in the same study, Holladay and colleagues proposed an alternative method where rat bone-marrow-derived MSCs were implanted in scaffolds loaded with IL-10 plasmid–polymer complexes, or “polyplexes”, allowing in vivo transfection [79]. This approach increased the MSCs’ retention rates for up to 21 days, which led to prolonged IL-10 release and decreased number of inflammatory cells. Although the idea of scaffold loading with bioactive molecules is not a new one [80], their delivery remains a challenge and ranges from chemical [81,82] or physical [83] incorporation to the use of micro- [84,85,86] or nanoparticles [87]. In this light, ECM as a natural source of bioactive molecules, such as VEGF [88], granulocyte-macrophage colony-stimulating factor (G-MCSF) [66], hepatocyte growth factor (HGF) [66,88], transforming growth factor-beta (TGFβ) [66,88], interleukin 3 (IL-3) [66], or interferon-gamma (INFγ) [66], is of great interest. Moreover, while state-of-the-art approaches suggest EVs’ integration into scaffolds as a powerful tool for cell signaling [89,90,91], ECM-based scaffolds are a natural source of matrix-bound nanovesicles (MBVs), a subgroup of EVs known for their unique immunomodulatory properties [92].

## 5. Cell-Free Approaches to Cartilage Repair: Targeting Inflammation

**Figure 1:**
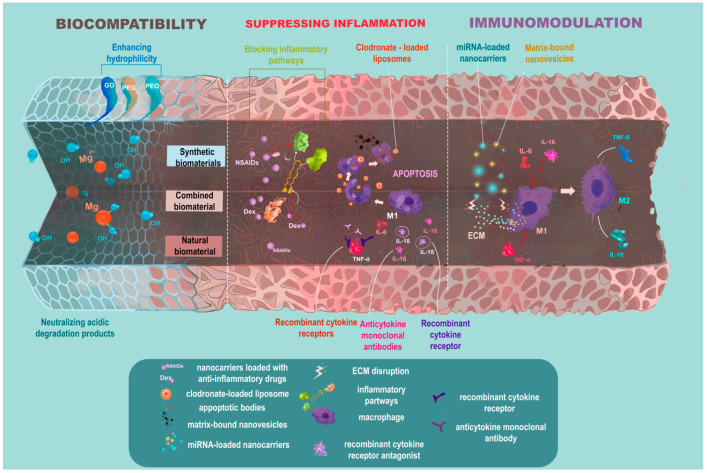
Evolution of the biocompatibility paradigm in tissue engineering. Excessive post-implantation inflammation is a common adverse effect in tissue engineering, and therefore biocompatibility is the cornerstone for constructs applied in tissue regeneration. Various strategies enhancing the construct’s biocompatibility have been proposed, including the use of natural biomaterials or functionalizing synthetic biomaterials in a way that would neutralize their degradation products or enhance their hydrophilicity. Yet, there is a trend of changing the paradigm of bioinert tissue constructs to bioactive ones. Thus, not only should they be biocompatible, but they should also be functionalized in a way that would target inflammation. Different strategies suppressing inflammation such as blocking inflammatory pathways or inducing inflammatory cells’ apoptosis have been proposed. However, state-of-the-art approaches suggest immunomodulation rather than suppressing inflammation for successful tissue remodeling and regeneration. In this light, miRNAs known for their unique ability to regulate multiple processes including macrophage polarization are of great interest as therapeutic agents.

Until quite recently, blocking the inflammatory pathways in OA was expected to be the cure for the disease; however, both anti-cytokine therapy [39,40,41,42,43,44,45] and macrophage depletion in vivo [32] have shown modest results. At present, complete evasion of inflammation is not considered beneficial for the disease outcome [93]. On the contrary, it is generally agreed upon that a moderate, controlled inflammatory reaction is essential for tissue remodeling and successful regeneration [94,95]. Therefore, there is a trend of changing the paradigm of “immune-evasive” bioinert tissue constructs to “immune-interactive”, bioactive ones [96], and enhancement of their immunomodulatory potential (Figure 1).

Paradoxically, the quest for “tunable” inflammation might lead to reconsideration of cell-based therapies, including MSC-based ones. Despite promising results in various fields of medicine, one important issue concerning their applicability in addition to those discussed in previous sections is their unpredictable behavior in vivo [97,98]. Not only are MSCs reported to become more immunogenic in the process of differentiation, but also to exhibit diametrically opposite immunoregulatory properties depending on the microenvironment. There is evidence that MSCs’ immunosuppressive effect is exerted only in the case of strong inflammation, while low inflammatory signals reduce MSCs’ immunosuppressive capacities and can even lead to MSC-mediated immune system activation [99], which is critical in treating low-grade inflammatory diseases, such as OA. Although MSCs’ plasticity is of great interest and warrants further study, cell-free approaches seem more appropriate for development of controllable therapeutic approaches at the moment.

While host immune response remains the fundamental problem in tissue engineering, inflammatory microenvironment, such as in OA joint, makes tissue regeneration even more challenging: speaking of scaffolds, not only should they be biocompatible, but they should also be functionalized in a way that would target already existing inflammation [100]. Although clinical trials are lacking, inflammation-targeting scaffolds for treating cartilage defects have demonstrated promising results in vivo (Table 1).

For example, a chitosan-based hydrogel with alginate-chitosan beads was reported to significantly reduce synovial inflammation in OA rabbit models for 6 weeks [101]. At the same time, an electrospun polylactic acid/gelatin-based scaffold functionalized with chondroitin sulfate was shown to downregulate inducible nitric oxide synthase (iNOS) and prostaglandin E synthase (PGES) in OA rabbit models for 12 weeks [102].

Known for their antioxidant properties, polyphenols such as tannins, curcumin, and resveratrol are extensively researched for treating multiple diseases, including OA [108]. Resveratrol was used by Wang and colleagues in the atelocollagen-based hydrogel system with polyacrylic acid, and significantly downregulated IL-1β, MMP-13, and cyclooxygenase-2 (COX-2) in OA rabbits for 6 weeks [103]. Li and colleagues have demonstrated that even an extract of a silk/graphene oxide-based scaffold modified with tannic acid/Sr2+ coating downregulated IL-6, IL-8, and MMPs in a model of papain-induced OA in rats for up to 4 weeks [104]. 

Dong and colleagues opted for dexamethasone as the primary inflammation-targeting agent [105]. They proposed a hydrogel based on catechol-modified gelatin and dopamine-modified oxidized hyaluronic acid and functionalized it with dexamethasone-loaded dendritic mesoporous organic silica nanoparticles. This system was reported to downregulate IL-6 and TNF-α in a rat OA model for 8 weeks [105].

Speaking about longer terms of follow-up, alginate hydrogel supplied with recombinant adeno-associated virus (rAAV)-associated IGF-I insulin-like growth factor 1 was reported to downregulate IL-1β and TNF-α in an OA model on minipigs for 1 year [106].

A recent study by Jia and colleagues was the first one to focus on macrophage polarization while treating OA with hydrogel in vivo [107]. The authors reported poly (salicylic acid)-F127-poly (salicylic acid) and hyaluronic acid-3-hydroxyanthranilic acid-based hydrogel not only to reduce synovial inflammation and downregulate iNOS in the synovium, but also to shift macrophage polarization towards the anti-inflammatory M2 phenotype.

EVs, used either to functionalize the scaffolds or alone, are of interest not only as a natural source of bioactive molecules but also as a unique vehicle for targeted therapies (Table 2). Their high biological activity is mainly determined by nucleic acids, namely miRNAs. Some estimates suggest that miRNAs potentially regulate more than 60% of human protein-coding genes [109].

In order to study the precise effect of a particular miRNA, EVs can be additionally loaded with different miRNAs. Another approach suggests blocking or downregulating miRNAs of interest to assess their contribution to any particular process. Thus, downregulating miR-26a-5p in human SMSCs demonstrated that hSMSC-derived EVs carrying miR-26a-5p could downregulate TNF-α and upregulate IL-10 in a rat OA model for 4 weeks [110]. On the other hand, human bone marrow mesenchymal stem cell (hBMSC)-derived EVs loaded with miR-26a-5p via lentiviral vector transduction were reported to reduce inflammatory cell infiltration in the synovium of OA rats for 8 weeks [111].

Similarly, hBMSC-derived EVs loaded with miR-361-5p via electroporation downregulated iNOS, MMP-3, MMP-13, IL-18, IL-6, and TNF-α in OA rats’ synovial tissues for 8 weeks [112]. At the same time, miR-31-loaded EVs derived from lentiviral-vector-transduced culture of hSMSCs downregulated IL-1β, IL-6, and TNF-α expression in a murine OA model for 12 weeks [113]. While many researchers choose human MSCs as the EVs’ source for OA treatment, some groups opt for allogenic cells for in vivo studies. Zhou [114] and Lai [115] chose primary rat synovial fibroblasts as the EVs’ source for rat OA models. The groups studied miR-126-3p [114] and miR-214-3p-loading [115], and both reported reduced synovial inflammation and downregulation of IL-1β and TNF-α for 10 weeks. Ye and colleagues transfected murine BMSCs with miR-3960 and reported the derived EVs to downregulate IL-6 and TNF-α in the serum of OA mice [116].

Both scaffolds and EVs are a source of inspiration for designing different nanomaterials and nanocarriers for treating various pathologic conditions [117,118,119,120], including OA (Table 3).

For example, chitosan-modified molybdenum disulfide nanosheets loaded with dexamethasone were reported to downregulate IL-1β, IL-8, and TNF-α in a murine OA model for 4 weeks [121]. Chitosan was also used in designing thermo-responsive nanospheres in a study by Kang and colleagues [122]. Loaded with kartogenin and diclofenac, these nanospheres were reported to significantly decrease the levels of COX-2 in the synovium of OA rats for up to 8 weeks. At the same time Jung and colleagues reported poly(d,l-lactide-co-glycolide) nanoparticles loaded with diacerein to suppress the blood levels of a variety of proinflammatory cytokines in OA rats for up to 9 weeks after a single intra-articular injection [123]. Some nanocarriers act as therapeutic agents themselves without any extra loading: for example, hollow MnO_2_ nanoparticles modified with NH2-PEG-NH2 downregulated the levels of IL-1β and IL-6 in the serum of OA mice for 8 weeks [124]. While most researchers use intra-articular injections when administering EV-based or nanocarrier-based OA treatment, Pei and colleagues reported reduced synovial inflammation in OA rats for 3 weeks following an intra-venous injection of polyhydroxylated fullerol nanoparticles [125]. Reduced synovial inflammation as well as IL-1β downregulation in the synovial fluid of OA mice were also reported for polyethylenimine conjugated with chondrocyte-affinity peptide and loaded with anti-Hif-2α siRNA [126]. Kim and colleagues reported methoxy poly(ethylene glycol)-b-poly (D,L-lactide) and PLGA-based nanoparticles loaded with the cytoprotective drug rebamipide to reduce the blood levels of IL-1β, IL-6, TNF-α, MMP-3, MMP-13, and COX-2 in OA rats [127].

Two recent studies focused on macrophage polarization modulation via functionalized nanoparticles. Bilirubin grafted polylysine nanoparticles loaded with IgG and the anti-inflammatory agent berberine were reported to reduce synovial inflammation, downregulate TNF-α, and decrease the M1/M2 ratio in the synovium of OA rats [128]. Resolvin D1-loaded nanoliposomes were equally reported to reduce the M1/M2 ratio in a murine OA model [129].

## 6. Matrix-Bound Nanovesicles: A Promising Therapeutic Agent 

EVs discussed in the previous section are exclusively liquid-phase vesicles, i.e., microvesicles or exosomes. However, MBVs bound to the ECM fibers can also serve as a source of regulatory miRNAs. It seems that there is no full overlap of liquid-phase EV and MBV functions due to the differences in their miRNA composition: for example, more than 50% of miRNAs were found to be differentially expressed in MBVs compared to liquid-phase EVs in mouse embryonic fibroblasts NIH 3T3 [130]. Inter alia, Hussey and colleagues reported miR-27a-5p to be upregulated in MBVs compared to liquid-phase EVs, which might contribute to their immunomodulatory potential: miR-27a-5p is known to exert negative regulation of NF-κB transcription factor activity, thus downregulating the proinflammatory IL-1β signaling pathway [131]. Moreover, being enriched with polyunsaturated fatty acids, MBVs seem to serve as a source of signaling lipid mediators, regulating inflammation-related pathways [130].

MBVs were reported to switch macrophages’ phenotype from proinflammatory M1 to anti-inflammatory M2 in vitro [92]. Furthermore, the fact that MBVs are embedded within the ECM as long as it is intact and are detached from the fibers ready for the cellular uptake in case of ECM disruption could be associated with their superior regenerative properties. Should this hypothesis be confirmed, it might mean no need for extra loading of MBVs with miRNAs when used as a therapeutic agent. 

While there are no studies yet evaluating MBVs’ effect in OA animal models, MBVs were reported to mitigate both acute and chronic RA in vivo [132]. The authors reported MBVs’ therapeutic efficacy to be equal to that of methotrexate and associated this effect with modulation of local synovial macrophages. 

ECM-based scaffolds and hydrogels successfully used in tissue engineering should probably contain MBVs, but to what extent MBVs contribute to their regenerative potential is yet to be confirmed. On the other hand, it was reported that short-term enzyme treatment of cartilage defects could improve the tissue-engineered constructs integration [133,134,135], which was associated with facilitated cell migration. However, enzyme treatment could also lead to the detachment and cellular uptake of MBVs, stimulating the healing of the tissue. 

## 7. Conclusions

Multiple strategies for OA management have been proposed, but the cure is yet to be found. One of the latest concepts suggests that OA treatment should focus on both cartilage repair and immunomodulation. This dual targeting can be achieved via the use of functionalized biomaterials, native or engineered nanovesicles, or else the combination of all these techniques. In view of the foregoing, mimicking natural processes of regeneration but enhancing them with the help of tissue engineering approaches or nanotechnologies seems to be the winning strategy.

## Figures and Tables

**Table 1:** Inflammation-targeting scaffolds for treating cartilage defects in vivo.

Scaffold	Primary Agent Targeting Inflammation	Animal Model	Defect Type	Treatment	Follow-Up	Effect on Inflammation	Assessment Method	Ref.
chitosan-based hydrogel with alginate-chitosan beads	chitosan	rabbit	ACLT	single intra-articular injection 1 week after the defect formation	6 weeks	reduced synovial inflammation	H&E staining	[101]
electrospun polylactic acid/gelatin-based scaffold functionalized with chondroitin sulfate	chondroitin sulfate	rabbit	chondral defect (3 mm diameter and 4 mm depth)	Immediate scaffold implantation	12 weeks	iNOS ↓ PGES ↓ in the joint tissues	IHC staining	[102]
atelocollagen-based hydrogel with polyacrylic acid and resveratrol	resveratrol	rabbit	chondral defect (4 mm diameter and 4 mm depth)	immediate scaffold implantation	2, 4, 6 weeks	IL-1β ↓ MMP-13 ↓ COX-2 ↓ in the joint tissues	qRT-PCR	[103]
silk/graphene oxide-based scaffold modified with tannic acid/Sr2+ coating	tannic acid	rat	papain- induced OA	intra-articular injection of the scaffold extract from day 10 every 5 days	4 weeks	IL-6 ↓ IL-8 ↓ MMPs ↓ in the meniscus and cartilage tissue	RT-PCR	[104]
catechol-modified gelatin and dopamine-modified oxidized hyaluronic acid-based hydrogel with Fe[3]^+^ and dendritic mesoporous organic silica nanoparticles	dexamethasone	rat	osteochondral defect (3.5 mm diameter and 5 mm depth)	single intra-articular injection	8 weeks	TNF-α ↓ IL-6 ↓ in the joint tissues	IHC staining	[105]
alginate hydrogel supplied with rAAV- IGF-I	IGF-I	minipig	chondral defect (4 mm diameter)	immediate scaffold implantation	1 year	IL-1β ↓ TNF-α ↓ in the joint tissues	IHC staining	[106]
poly (salicylic acid)-F127-poly (salicylic acid) and hyaluronic acid-3-hydroxyanthranilic acid-based hydrogel	3-hydroxyanthranilic acid	rat	papain- induced OA	intra-articular injections once per week	3, 6 weeks	reduced synovial inflammation iNOS ↓ M1 ↓ M2 ↑ in the synovium	H&E- staining IHC staining	[107]

ACLT—anterior cruciate ligament transection, COX-2—cyclooxygenase-2, H&E—hematoxylin and eosin, IGF-I—insulin-like growth factor 1, IHC—immunohistochemistry, iNOS—inducible nitric oxide synthase, M1—proinflammatory macrophages, M2—anti-inflammatory macrophages, PGES—prostaglandin E synthase, qRT-PCR—quantitative real-time polymerase chain reaction, rAAV—recombinant adeno-associated virus, Ref.—reference, RT-PCR—reverse transcription-polymerase chain reaction.

**Table 2:** Inflammation-targeting EVs for treating cartilage defects in vivo.

EV Source	miRNA Studied	EV loading Method	Animal Model	Defect Type	Treatment	EV Dose	Follow-Up	Effect on Inflammation	Assessment Method	Ref.
hSMSCs	miR-26a-5p	-	rat	ACLT, MCLT, MMT,	intra-articular injections on day 7, 14, 21	10^11^ EVs/mL, 30 μL	4 weeks	TNF-α ↓ IL-10 ↑ in the joint tissues	ELISA	[110]
hBMSCs	miR-26a-5p	lentiviral vector transduction	rat	ACLT, meniscectomy	intra-articular injections for 7 days post-surgery	250 ng/5 µL	8 weeks	reduced number of inflammatory cells IL-1β ↓ in serum	H&E staining ELISA	[111]
hBMSCs	miR-361-5p	EV electroporation	rat	ACLT	intra-articular injections for 7 days post-surgery	250 ng/5 µL	8 weeks	iNOS ↓ MMP-3 ↓ MMP-13 ↓ IL-18 ↓ IL-6 ↓ TNF-α ↓ in the synovial tissues	Western blot	[112]
hSMSCs	miR-31	lentiviral vector transduction	mouse	ACLT, MCLT, MMT	intra-articular injection every 3 days for 4 weeks	5 μL/mL	12 weeks	IL-1β ↓ IL-6 ↓ TNF-α ↓ in the synovial fluid	ELISA	[113]
primary rat synovial fibroblasts	miR-126-3p	cell culture transfection	rat	ACLT, MMR	intra-articular injection once per week from the 4th week post-surgery	500 μg/mL, 40 μL	10 weeks	reduced synovial inflammation IL-1β ↓ TNF-α ↓ in the cartilage	H&E- staining IHC staining	[114]
primary rat synovial fibroblasts	miR-214-3p	cell culture transfection	rat	ACLT, MMR	intra-articular injection once per week from the 4th week post-surgery	not specified	10 weeks	reduced synovial inflammation IL-1β ↓ TNF-α ↓ in the cartilage and synovium	H&E- staining IHC staining	[115]
mouse BMSCs	miR-3960	cell culture transfection	mouse	MCLT, MMT	intra-articular injection once per week for 3 weeks	100 μg/mL,10 μL	7 weeks	IL-6 ↓ TNF-α ↓ in the serum	ELISA	[116]

ACLT—anterior cruciate ligament transection, H&E—hematoxylin and eosin, hBMSCs—human bone marrow mesenchymal stem cells, hSMSCs—human synovial mesenchymal stem cells, IHC—immunohistochemistry, iNOS—inducible nitric oxide synthase, MCLT—medial collateral ligament transection, MMR—medial meniscus resection, MMT—medial meniscus transection, Ref.—reference.

**Table 3:** Inflammation-targeting nanocarriers for treating cartilage defects in vivo.

Nanocarrier	Loaded Agents	Animal Model	Defect Type	Treatment	Follow-Up	Effect on Inflammation	Assessment Method	Ref.
chitosan-modified molybdenum disulfide nanosheets	dexamethasone	mouse	papain- induced OA	intra-articular injections every 3 days; near-infrared light exposure	4 weeks	IL-1β ↓ IL-8 ↓ TNF-α ↓ in the synovium	IHC staining	[121]
thermo-responsive chitosan oligosaccharide nanospheres conjugated with pluronic F127 grafting carboxyl group	kartogenin diclofenac	rat	ACLT, MM destabilization	intra-articular injections at weeks 7 and 10	8 weeks	COX-2 ↓ in the synovium	ELISA	[122]
poly(d,l-lactide-co-glycolide) nanoparticles	diacerein	rat	MIA- induced OA	single intra-articular injection	9 weeks	IL-1 ↓ IL-6 ↓ TNF-α ↓ MMP-3 ↓ MMP-13 ↓ COX-2 ↓ ADAMTS-5 ↓ IL-4 ↑ IL-10 ↑ in the whole blood	real-time PCR	[123]
hollow MnO_2_ nanoparticles modified with NH2-PEG-NH2	-	mouse	MM destabilization	intra-articular injections 3 times a week for 4 weeks	8 weeks	IL-1β ↓ IL-6 ↓ in the serum	ELISA	[124]
polyhydroxylated fullerene C60 (fullerol) nanoparticles	-	rat	MIA- induced OA	single intra-venous injection	3 weeks	reduced synovial inflammation	H&E staining	[125]
polyethylenimine conjugated with chondrocyte-affinity peptide	anti-Hif-2α siRNA	mouse	ACLT and MCL dissection	weekly intra-articular injections	7 weeks	reduced synovial inflammation IL-1β ↓ in the synovial fluid	H&E stainingELISA	[126]
methoxy poly(ethylene glycol)-b-poly (D,L-lactide) and PLGA-based nanoparticles	rebamipide	rat	MIA- induced OA	single intra-articular injection	4, 8 weeks	IL-1β↓ IL-6↓ TNF-α↓ MMP-3↓ MMP-13↓ COX-2↓ in the whole blood	real-time PCR	[127]
bilirubin grafted polylysine nanoparticles	IgG, berberine	rat	ACLT	intra-articular injections on day 35, 40, 45, 50, 55, and 60	65 days	reduced synovial inflammation TNF-α ↓ M1/M2 ratio ↓ in the synovium	H&E stainingIHC	[128]
nanoliposomes	resolvin D1	mouse	MM destabilization	intra-articular injections at weeks 1, 4, and 8	3 months	M1/M2 ratio ↓ in the synovium	IHC	[129]

ACLT—anterior cruciate ligament transection, anti-Hif-2α—hypoxia-inducible factor-2α, H&E—hematoxylin and eosin, IHC—immunohistochemistry, M1—proinflammatory macrophages, M2—anti-inflammatory macrophages, MCL—medial collateral ligament, MIA—monosodium iodoacetate, MM—medial meniscus, Ref.—reference.

## Data Availability

Not applicable.

## Abbreviations

ACI—autologous chondrocyte implantation; BMP2—bone morphogenic protein 2; COX-2—cyclooxygenase-2; DAMPs—damage/danger-associated molecular patterns; dECM—decellularized extracellular matrix; DMOADs—disease-modifying osteoarthritis drugs; ECM—extracellular matrix; EVs—extracellular vesicles; G-MCSF—granulocyte-macrophage colony-stimulating factor; GO—graphene oxide; HA—hyaluronic acid; HGF—hepatocyte growth factor; HMGB1—high-mobility group protein B1; HPMAm-lac—hydroxypropyl methacrylamide mono- and dilactate; IGF-I—insulin-like growth factor 1; IL—interleukin; INFγ—interferon gamma; iNOS—inducible nitric oxide synthase; MACI—matrix-induced autologous chondrocyte implantation; MBVs—matrix-bound nanovesicles; MMPs—matrix metalloproteinases; MSCs—mesenchymal stromal cells; NF-κB—nuclear factor kappa-light-chain-enhancer of activated B cells; NLRP3—NOD-, LRR-, and pyrin domain-containing protein 3; NSAIDs—non-steroidal anti-inflammatory drugs; OA—osteoarthritis; OATS—osteochondral autograft transfer system; PCL—polycaprolactone; PEG—polyethylene glycol; PEO—polyethylene oxide; PGA—poly(glycolic acid); PGES—prostaglandin E synthase; PLA—polylactide; PLGA—poly(lactic-co-glycolic acid); PRRs—pattern recognition receptors; RA—rheumatoid arthritis; rAAV—recombinant adeno-associated virus; TGFβ—transforming growth factor beta; TLRs—Toll-like receptors; TNF-α—tumor necrosis factor alpha; VEGF—vascular endothelial growth factor.
